# Evaluation of Antiasthmatic Activity of *Curculigo orchioides* Gaertn. Rhizomes

**DOI:** 10.4103/0250-474X.44590

**Published:** 2008

**Authors:** Pranali Pandit, Anita Singh, A. R. Bafna, P. V. Kadam, M. J. Patil

**Affiliations:** Padmashree Dr. D. Y. Patil Institute of Pharmaceutical Sciences and Research, Pimpri, Pune-411 018, India; 1 Marathwada Mitra Mandal’s College of Pharmacy, Thergaon (Kalewadi), Pune-411 033, India

**Keywords:** Antiasthmatics, bronchoconstriction, *Curculigo orchioides*, eosinophils

## Abstract

The ethanol extract of *Curculigo orchioides* was evaluated for antiasthmatic activity by using various *in vitro* and *in vivo* animal models. *In vitro* models like isolated goat tracheal chain preparation and isolated guinea pig ileum preparation were studied to know basic mechanism by which extract shows relaxant activity. The study showed that extract is effective against histamine-induced contraction. In isolated goat tracheal chain preparation and isolated guinea pig ileum preparation extract exhibits maximum relaxant effect (p< 0.01) against histamine at concentrations 100μg/ml and 25μg/ml respectively. Animal studies involved use of histamine induced bronchoconstriction in guinea pigs, egg albumin induced passive paw anaphylaxis in rats and haloperidol-induced catalepsy in mice. These studies showed significant (p< 0.01) protection at lower doses while further increase in the dose level showed reduced activity. Biochemical estimations in milk-induced total leukocytes count and milk-induced differential leukocyte count were also studied. In this study there was maximum increase in leucocytes and lymphocytes (99%) and maximum decrease in eosinophils up to 0% at dose 375mg/kg p.o. body weight was observed. The results of these studies indicated usefulness of ethanol extract of *Curculigo orchioides* in asthma.

Asthma is a very commonly occurring condition that is most difficult to control in chronic stage. In the United States alone, asthma affects almost 17 million people, and this is a 75% increase in the last 20 years. This means that about 1 out of every 20 adults and close to 1 out of 13 children today have asthma. An alarming fact is that since 1980, asthma in children under age 5 has risen remarkably. In school age children, asthma has risen by 75%. India alone has an estimated 15–20 million asthmatics. Mortality data from developed countries show that the rates varies from 0.1- 0.8 per 100,000 persons aged 5–34^1^. For managing asthma attacks, symptomatic relief is foremost requirement. In India, in various traditional systems like Ayurveda, Unani and Siddha, numerous herbs were mentioned for therapeutic use in asthma. *Curculigo orchioides* Gaertn. is one of the important plant mentioned in Ayurveda and Unani for asthma^2^,^3^. In Chinese medicinal system also, *Curculigo orchioides* is one of the important plant. Its rhizomes were used as aphodisiac, alterative, appetizer, fattening; useful in piles, jaundice and colic disorders. Methanol extract has shown immunostimulant^4^, antioxidant^5^, hepatoprotective and antiinflammatory activity^6^.

Triterpenoides (Curculigol), glycosides (Curculignin A, B, C) curculigosaponin such as curculigenin A, B, C, corchicoside A, curculigoside B and alkaloids (Yuccagenin, Lycorin) are major components reported which might be responsible for various medicinal use of herb 7–17. Although, there is no scientific proof of the efficacy of plant extracts for antiasthmatic activity, the aim of this study was to evaluate antiasthmatic effect of *Curculigo orchioides*.

## MATERIALS AND METHODS

Rhizomes of *Curculigo orchioides* were collected from local market of Pune, MS, India and were identified and authenticated from Agharkar Research Institute, Pune, India. The voucher specimen was deposited bearing no. AHMA- R-065. The collected material was air dried at 35–40° and pulverized in electric grinder. The powder obtained was extracted in ethanol by using Soxhlet extractor. The yield obtained was 6.45% w/w. Phytochemical screening reveled presence of phenolic compounds, flavanoids, tannins, glycosides, alkaloids saponnis and carbohydrates^18^. The extract was stored in a refrigerator for further use. Dunkon-Hartley Guinea pigs weighing 350–400g of either sex brought from National toxicological center Pune, Wistar rats weighing 150–250 g and Swiss mice of either sex bread at the Padmashree Dr. D. Y. Patil Institute of Pharmaceutical Sciences and Research, Pimpri, Pune were housed at standard conditions of temperature (22±1°) and 12/12 h light/dark cycle. They were fed with standard pellet diet (Amrut laboratory animal feed, Sangli, India) and had free access to water. Permission for conduct of these experiments was obtained from Institutional Animal Ethical Committee (IAEC). Toxicity studies conducted as per internationally accepted protocol drawn under OECD guidelines 420 in Swiss mice at a dose level of extracts up to 10 g/kg.

### Isolated goat tracheal chain preparation^19^:

Isolated adult goat tracheal tissue was obtained from slaughter house. Trachea was cut into individual rings and tied together in series to form a chain. Trachea was suspended in bath of Kreb’s solution and was continuously aerated at 37±0.5°. Dose response curves of histamine in plain Kreb’s solution and in Kreb’s solution containing 100 μg/ml *Curculigo orchioides* extract were performed. Percent of maximum contractile responses were plotted to record dose response curves of histamine in the absence and presence of plant extract.

### Isolated guinea pig ileum preparation^20^:

The guinea pigs (overnight fasted) were sacrificed and the ileum was mounted in an organ bath containing Tyrode solution, which was continuously aerated at 37±0.5°. Dose response curve of histamine in plain Tyrode solution and in Tyrode solution containing 25 μg/ml *Curculigo orchioides* extract were performed. Percentage maximum contractile response was plotted to generate dose response curve of histamine, in the absence and presence of the plant extract.

### Histamine induced bronchoconstriction in guinea pigs^21^:

Duncon-Hartley Guinea pigs were divided into 8 groups (n=6), control group received saline and other groups received single dose of extract (75, 150, 200, 300, 600, 1200 mg/kg p.o.) respectively. Chlorpheniramine maleate (2 mg/kg) was used as positive control. Prior to and after drug treatment each animal was placed in the histamine chamber and exposed to 0.2% histamine aerosol. The preconvulsive time (PCT) was determined from the time of exposure to onset of dyspnoea leading to the appearance of preconvulsive dyspnoea in a min. The% protection offered by drugs in pCT 0 was calculated for each dose and positive control.

### Haloperidol-induced catalepsy^22^:

Swiss mice were divided into 8 groups (n=6), control group received saline and other groups received single dose of extract (125, 250, 375, 500, 1000, 2000 mg/kg p.o.), respectively. chlorpheniramine maleate (10 mg/kg) was used as positive control. All the group received haloperidol (1 mg/kg i.p.) 1 h after the drug administration and the duration of catalepsy was measured at 0, 30, 60, 90, 120 and 150 min.

### Passive paw anaphylaxis in rats^23^:

Wistar rats were given subcutaneously thee doses of 100 μg of egg albumin on d 1, 3 and 5. On day 10 of sensitization, blood was collected and centrifuged to separate serum. Animals were divided into eight groups (n=6). Control group received saline and other groups received single dose of extract 85, 175, 250, 350, 700, 1400 mg/kg p.o. Dexamethasone was used as standard (0.27 mg/kg p.o). Prior to drug treatment animals were sensitized with serum. Next 24 h, after drug treatment animals again challenged with 10 μg egg albumin and edema inhibition was calculated.

### Milk-induced leucocytosis in mice^24^,^25^:

Swiss mice were divided into 8 groups (n=6), Control group received saline other groups received single dose of extract (125, 250, 375, 500, 1000, 2000 mg/kg p.o.), respectively. Only Milk received group served as an intoxicant. After 1 h of drug treatment except control all groups received boiled and cooled milk injection in dose of (4 ml/kg s.c.). Total leukocyte count was done in each group before drug administration and 24 h after milk injection.

## RESULTS AND DISCUSSION

The present study dealt with screening of antiasthamatic activity of ethanol extract of rhizomes of *Curculigo orchioides* Gaertn. Bronchial asthma is a chronic inflammatory disease, characterized by both bronchoconstriction and airway inflammation which leads to bronchial hyper responsiveness to various stimuli, in which many cell types play a role, more important being mast cells, eosinophils and T-lymphocytes.

Different agonists like acetylcholine, histamine, 5-hydroxyltryptamine and bradykinin are responsible for contractile responces^26^. In isolated goat tracheal chain and isolated guinea pig ileum preparation, there is a right side shift of dose response curve of histamine in the presence of ethanol extract of *Curculigo orchioides* indicating antiasthmatic action (Table 1).

**TABLE 1:** EFFECT OF THE ETHANOL EXTRACT OF *CURCULIGO ORCHIOIDES* ON HISTAMINE-INDUCED CONTRACTIONS

Dose of histamine	Isolated goat tracheal chain preparation		Isolated guinea pig ileum preparation	
		
(2.5 μg/ml)	Control group	Test group	Control group	Test group
(ml)	% Maximum contraction	% Maximum contraction	% Maximum contraction	% Maximum contraction
0.1	25.758±2.579	14.386±1.162*	17.776±0.925	12.058±1.188*
0.2	51.074±3.971	28.418±1.874*	31.748±0.501	22.534±1.618*
0.4	72.278±1.790	44.91±2.968*	63.488±2.511	43.806±2.163*
0.8	89.822±1.291	54.731±3.010*	93.33±2.988	59.682±2.952*
1.6	91.578±2.444	63.856±5.564*	93.33±2.988	64.446±1.384*

Effect of the ethanol extract of *Curculigo orchioides* (co) on histamine-induced contraction on the isolated goat tracheal chain preparation and the isolated guinea pig ileum preparation was tabulated. All values are expressed as mean±SEM of a sample size of n=6; level of significance chosen was

* p<0.05. All treated groups were compared with control group.

Histamine is one of the major inflammatory mediators in the immediate phase of asthma, causing airway hyper responsiveness and bronchial airway inflammation. The study regarding involvement of H1 and H2 receptors has been done in experimental asthma in guinea pig using respiratory smooth muscle and it was confirmed that there is prominent involvement of H1 receptors as compared to H2 receptors especially in asthama^26^.

The maximum percentage protection i.e. 90.11% observed at 200mg/kg dose for bronchorelaxant study comparable with that of standard chlorpheniramine maleate 91.92%. Statistical significance in post treated exposition time and mean exposition time also showed 200 mg/kg as effective (p< 0.01) dose. Further increase in the dose showed decreased activity (Table 2).

**TABLE 2:** EFFECT OF THE ETHANOL EXTRACT OF *CURCULIGO ORCHIOIDES* (CO) ON HISTAMINE-INDUCED BRONCHOCONSTRICTION

Groups	Dose in mg/kg p.o.	PCT (Before) T_1_	PCT (After) T_2_	Mean exposition time	% Protection
1	Control	1.488±0.104	1.504±0.187	0.076± 0.138	1.06
2	75 CO	1.144±0.0314	2.67±0.237	1.526±0.2075*	57.154
3	150 CO	1.278±0.1044	3.436±0.187	2.158±0.138*	62.81
4	200 CO	1.258±0.0753	12.716±0.934	11.458±0.884*	90.11
5	300 CO	1.356±0.0532	5.58±0.240	4.232±0.264*	75.734
6	600 CO	1.432±.0519	4.208±0.306	2.776±0.3166*	65.969
7	1200 CO	1.29±0.152	1.382±0.088	0.272±0.0985	6.66
8	CPM (2 mg/kg)	1.308±0.1007	14.81±0.19	13.614±0.1607*	91.92

All values are expressed as mean±SEM of a sample size of n=6; level of significance chosen was *p<0.05. All treated groups were compared with control group. CPM is chlorpheniramine maleate (2 mg/kg)

Haloperidol induces catalepsy by inhibiting dopamine D2 receptors and inhibits dopamine secretion. Dopamine is agonist for adrenaline. Adrenaline is physiological antagonist of histamine. So as there decrease in dopamine there is imbalance in neurotransmitters means high level of histamine^27^. In this study significant (p< 0.01) protection against haloperidol-induced catalepsy at dose 375 mg/kg. Further increase in the dose showed decreased activity (Table 3)

**TABLE 3:** EFFECT OF THE ETHANOL EXTRACT OF *CURCULIGO ORCHIOIDES* (CO) ON HALOPERIDOL-INDUCED CATALEPSY

Group	Dose mg/kg	Duration of catalepsy (sec) at Mean±SEM				
		
		30 min	60 min	90 min	120 min	150 min
1	Control	236.66±12.82	273.66±8.42	285.33±4.72	299.66±0.33	238.66±12.96
2	125 CO	226.00±0.68	254.5±2.21	234.0±1.34*	249.0±0.85*	225.6±1.563
3	250 CO	219.33±2.246	248.0±1.25*	218.50±2.2*	228.5±2.87*	210.0±2.14*
4	375 CO	112.0±4.93*	93.0±1.96*	70.83±2.76*	52.83±2.92*	72.0±1.88*
5	500 CO	190.1±13.5*	227.33±8.7*	205.0±10.3	208.0±5.2*	200.83±3.7*
6	1000 CO	219.6±7.1	236.3±9.68*	214.16±2.0*	228.6±7.9*	212.0±0.774
7	2000 CO	223.8±1.1	255.6±3.93	239.6±7.14*	246.6±0.84*	228.6±4.28
8	CPM (10 mg/kg)	99.16±5.68*	76.5±3.53*	62.16±2.71*	42.83±1.93*	63.5±4.87*

All values are expressed as mean±SEM of a sample size of n=6; level of significance chosen was * p<0.05. All treated groups were compared with control group. CPM is chlorpheniramine maleate (2 mg/kg)

Allergic asthama is a chronic inflammatory process occurring due to exposure of allergen resulting in the activation of T-lymphocyte with subsequent release of inflammatory mediators. Immuno-modulating agents are useful in the treatment of asthma by inhibiting the antigen-antibody (AG-AB) reaction and there by inhibiting the release of inflammatory mediators^27^. *Curculigo orchioides* has been reported to possess antiinflammatory activity^6^. Percent inhibition of paw edema volume was calculated and maximum effective dose was observed at 250 mg/kg. at different hour intervals it was found that effect of dose 250 mg/kg was maximum up to 24 h, further percent inhibition goes on decreasing. But still that percent inhibition in paw edema was significantly effective as compare to other doses. Where as, in statistical analysis of paw edema volume it was observed that 250 mg/kg dose had significant (p< 0.01) effect comparable that with dexamethasone. Here also observed that further increase in dose decreased activity (Table 4).

**TABLE 4:** EFFECT OF *CURCULIGO ORCHIOIDES* ETHANOL EXTRACT (CO) ON PASSIVE PAW ANAPHYLAXIS

Groups	Dose mg/kg	Paw Edema Volume (ml) Mean ± SEM			
		
		1 h	2 h	3 h	4 h
1	Control	0.90±0.0367	0.76±0.0396	0.63±0.0246	0.56±0.0178
2	85 CO	0.59±0.0881*	0.44±0.0779*	0.38±0.0545*	0.34±0.0386*
3	175 CO	0.75±0.0208*	0.57±0.0195*	0.44±0.0250*	0.41±0.0255*
4	250 CO	0.54±0.0499*	0.31±0.0478*	0.33±0.0402*	0.29±0.0418
5	350 CO	0.63±0.0225*	0.43±0.0155*	0.47±0.0110*	0.44±0.0129*
6	700 CO	0.61±0.0290*	0.49±0.0184*	0.50±0.108	0.47±0.0132
7	1400 CO	0.64±0.5050*	0.49±0.0292*	0.53±0.0225	0.49±0.0251
8	Dexamethasone (0.27 mg/kg)	0.45±0.0348*	0.27±0.0580*	0.29±0.0572*	0.25±0.0407*

All values are expressed as mean±SEM of a sample size of n=6; level of significance chosen was *p<0.05. All treated groups were compared with control group. CPM is chlorpheniramine maleate (2 mg/kg)

Herbal formulations used in the treatment of asthma include some antistress herbs to enable adoption to stress since excessive stress or nervous debility may aggravate symptoms of asthma. The normalization effect of an adaptogen can be observed in milk-induced leukocytosis (increase in leukocyte count) after parentral administration of milk. In the milk induced leukocytes count, expected results were to decrease leukocyte count at 375 mg/kg. But result obtained was exactly opposite i.e. at the dose 375 mg/kg there is highest increase in leukocyte count. (Table 5)

**TABLE 5:** EFFECT OF THE ETHANOL EXTRACT OF *CURCULIGO ORCHIOIDES* (CO) ON TOTAL LEUKOCYTE COUNT

Groups	Treatment	Number of leukocytes (Cu.mm.)		
		
		Before treatment	After treatment	Difference
1	Control (10 ml/kg saline)	6230±312.49	6550±287.23	320±155.4
2	CO 125 + Milk (4 ml/kg sc)	7340±545.07	8380±452.38	1040±344.02*
3	CO 250 + Milk (4 ml/kg sc)	5980±336.01	7270±410.51	1290±88.600*
4	CO 375 + Milk (4 ml/kg sc)	6730±449.05	9680±538.89	3350±383.73*
5	CO 500 + Milk (4 ml/kg sc)	7010±187.35	7270±1601.9	1880±308.06*
6	CO 1000 + Milk (4 ml/kg sc)	5530±196.60	7110±201.49	1700±226.38*
7	CO 2000 + Milk (4 ml/kg sc)	7400±135.09	8900±85.147	1500±130.38*
8	Saline (10ml/kg) + Milk (4 ml/kg sc)	6470±494.62	11460±776.76	4990±658.48 ^##^

All values are expressed as mean±SEM of a sample size of n=6; level of significance chosen was *p<0.05. All treated groups were compared with control group. CPM is chlorpheniramine maleate (2 mg/kg)

Most allergic and non-allergic asthmatics, including those with mild asthma, have bronchial eosinophilia and there is a significant association between eosinophil activation and asthma severity as well as bronchial hyper responsiveness^25^. Therefore differential leukocyte count was carried out for doses 125, 250, 375, 500, 1000, 2000 mg/kg p.o. body weight. The result showed significant (p <0.01) i.e. up to 0% decrease in eosinophils count at dose 250, 375, 500 mg/kg. But at dose 375 mg/kg there is tremendous i.e. up to 99% of lymphocytes count. (Table 6). It indicates that increased leukocyte count in total leucocytes count model, mainly due to increased lymphocyte (B and T cells) count.

**TABLE 6:** EFFECT OF THE ETHANOL EXTRACT OF *CURCULIGO ORCHIOIDES* (CO) ON DIFFERENTIA LEUKOCYTES COUNT

Groups	Treatment	Differential Leukocyte Count (%)		
		
		Neutrophils	Lymphocytes	Eosinophils
1	Control	06±0.707	88±1.342	02±0.3162
2	CO 125 + Milk (4 ml/kg sc)	05±0.316*	93±1.140	02±0.4472*
3	CO 250 + Milk (4 ml/kg sc)	05±0.447*	95±0.632*	00±0.0*
4	CO 375 + Milk (4 ml/kg sc)	01±0.316*	99±0.316*	00±0.0*
5	CO 500 + Milk (4 ml/kg sc)	03±0.316*	97±0.707*	00±0.0*
6	CO 1000 + Milk (4 ml/kg sc)	29±1.304*	70±1.844*	01±0.3162*
7	CO 2000 + Milk (4 ml/kg sc)	13±0.632*	83±1.342*	04±0.0
8	Saline (10 ml/kg) + Milk (4 ml/kg sc)	08±0.707	88±2.828	04±0.3162

All values are expressed as mean±SEM of a sample size of n=6; level of significance chosen was *p<0.05. All treated groups were compared with control group. CPM is chlorpheniramine maleate (2 mg/kg)

Drugs effective in asthma are mostly steroidal in nature. *Curculigo orchioides* extract contain steroidal nucleus in form of triterpenoides and various sapogenins and saponin glycosides. So antiasthmatic activity showed by *Curculigo orchioides* might be because of these chemical moieties.
